# Nutritional risk factors for SARS-CoV-2 infection: a prospective study within the NutriNet-Santé cohort

**DOI:** 10.1186/s12916-021-02168-1

**Published:** 2021-11-30

**Authors:** Mélanie Deschasaux-Tanguy, Bernard Srour, Laurent Bourhis, Nathalie Arnault, Nathalie Druesne-Pecollo, Younes Esseddik, Fabien Szabo de Edelenyi, Julien Allègre, Benjamin Allès, Valentina A. Andreeva, Julia Baudry, Leopold K. Fezeu, Pilar Galan, Chantal Julia, Emmanuelle Kesse-Guyot, Sandrine Péneau, Serge Hercberg, Nathalie Bajos, Gianluca Severi, Marie Zins, Xavier de Lamballerie, Fabrice Carrat, Mathilde Touvier, on behalf of the SAPRIS-SERO study group, Fabrice Carrat, Pierre-Yves Ancel, Nathalie Bajos, Marie-Aline Charles, Gianluca Severi, Mathilde Touvier, Marie Zins, Sofiane Kab, Adeline Renuy, Stephane Le-Got, Celine Ribet, Emmanuel Wiernik, Marcel Goldberg, Marie Zins, Fanny Artaud, Pascale Gerbouin-Rérolle, Mélody Enguix, Camille Laplanche, Roselyn Gomes-Rima, Lyan Hoang, Emmanuelle Correia, Alpha Amadou Barry, Nadège Senina, Gianluca Severi, Fabien Szabo de Edelenyi, Julien Allègre, Nathalie Druesne-Pecollo, Younes Esseddik, Serge Hercberg, Mathilde Touvier, Marie-Aline Charles, Pierre-Yves Ancel, Valérie Benhammou, Anass Ritmi, Laetitia Marchand, Cecile Zaros, Elodie Lordmi, Adriana Candea, Sophie de Visme, Thierry Simeon, Xavier Thierry, Bertrand Geay, Marie-Noelle Dufourg, Karen Milcent, Clovis Lusivika-Nzinga, Gregory Pannetier, Nathanael Lapidus, Isabelle Goderel, Céline Dorival, Jérôme Nicol, Fabrice Carrat, Cindy Lai, Hélène Esperou, Sandrine Couffin-Cadiergues, Jean-Marie Gagliolo, Hélène Blanché, Jean-Marc Sébaoun, Jean-Christophe Beaudoin, Laetitia Gressin, Valérie Morel, Ouissam Ouili, Jean-François Deleuze, Stéphane Priet, Paola Mariela Saba Villarroel, Toscane Fourié, Souand Mohamed Ali, Abdenour Amroun, Morgan Seston, Nazli Ayhan, Boris Pastorino, Xavier de Lamballerie

**Affiliations:** 1 Sorbonne Paris Nord University, Inserm U1153, Inrae U1125, Cnam, Nutritional Epidemiology Research Team (EREN), Epidemiology and Statistics Research Center – University of Paris (CRESS), Bobigny, France; 2 IRIS, UMR CNRS 8156, EHESS, Inserm U997, Aubervilliers, France; 3 Paris-Saclay University, UVSQ, Inserm, Gustave Roussy, “Exposome and Heredity” team, CESP UMR1018, Villejuif, France; 4 Department of Statistics, Computer Science and Applications “G. Parenti”, University of Florence, Florence, Italy; 5 Paris University, Paris, France; 6 Inserm UMS 11, Paris Saclay University, Villejuif, France; 7 Unité des Virus Emergents (UVE), Aix Marseille Univ, IRD 190, INSERM 1207, IHU Méditerranée Infection, Marseille, France; 8 Sorbonne Université, Inserm, Institut Pierre-Louis d’Epidémiologie et de Santé Publique, Paris, France; 9 Département de Santé Publique, APHP, Sorbonne Université, Paris, France

**Keywords:** SARS-CoV-2, Diet, Vitamins, Seroprevalence, Cohort study

## Abstract

**Background:**

Nutritional factors are essential for the functioning of the immune system and could therefore play a role in COVID-19 but evidence is needed. Our objective was to study the associations between diet and the risk of SARS-CoV-2 infection in a large population-based sample.

**Methods:**

Our analyses were conducted in the French prospective NutriNet-Santé cohort study (2009–2020). Seroprevalence of anti-SARS-CoV-2 antibodies was assessed by ELISA on dried blood spots. Dietary intakes were derived from repeated 24 h dietary records (at least 6) in the two years preceding the start of the COVID-19 pandemic in France (February 2020). Multi-adjusted logistic regression models were computed.

**Results:**

A total of 7766 adults (70.3% women, mean age: 60.3 years) were included, among which 311 were positive for anti-SARS-CoV-2 antibodies. Dietary intakes of vitamin C (OR for 1 SD=0.86 (0.75–0.98), *P*=0.02), vitamin B9 (OR=0.84 (0.72–0.98), *P*=0.02), vitamin K (OR=0.86 (0.74–0.99), *P*=0.04), fibers (OR=0.84 (0.72–0.98), *P*=0.02), and fruit and vegetables (OR=0.85 (0.74–0.97), *P*=0.02) were associated to a decreased probability of SARS-CoV-2 infection while dietary intakes of calcium (OR=1.16 (1.01–1.35), *P*=0.04) and dairy products (OR=1.19 (1.06–1.33), *P*=0.002) associated to increased odds. No association was detected with other food groups or nutrients or with the overall diet quality.

**Conclusions:**

Higher dietary intakes of fruit and vegetables and, consistently, of vitamin C, folate, vitamin K and fibers were associated with a lower susceptibility to SARS-CoV-2 infection. Beyond its established role in the prevention of non-communicable diseases, diet could therefore also contribute to prevent some infectious diseases such as COVID-19.

**Supplementary Information:**

The online version contains supplementary material available at 10.1186/s12916-021-02168-1.

## Background

First identified in December 2019, the severe acute respiratory syndrome coronavirus 2 (SARS-CoV-2) has massively spread worldwide, causing coronavirus disease 2019 (COVID-19), a disease ranging from asymptomatic and mild forms to severe forms requiring hospitalization and sometimes leading to death [1]. This paved the way to a vast array of research aiming to describe, understand, and predict various aspects of the COVID-19 pandemic. In particular, the identification of modifiable risk factors associated with the likelihood of being infected may provide some leverage for disease prevention, in parallel to vaccination programs.

Many hypotheses were raised regarding a putative role of nutrition in COVID-19, in the susceptibility to infection, in the severity of the disease and associated outcomes [2, 3]. The foods we eat indeed provide us with macronutrients, vitamins, minerals, and other bioactive compounds that are essential for a proper functioning of the immune system [2, 3]. Inadequate nutritional intakes could therefore impair the immune response and lead to a higher susceptibility to SARS-CoV-2 infection.

To our knowledge, two prospective studies have examined the prospective link between dietary intakes and the risk of SARS-CoV-2 infection: a Spanish study using a self-reported COVID-19 questionnaire without objective serology data, suggesting a potential protective association with a higher adherence to the Mediterranean diet [4]. Another study in the UK, used a simplified 17-item food questionnaire, in individuals who voluntarily sought testing by real-time polymerase chain reaction tests (RT-PCR), and suggested a protective association for higher intakes of coffee and vegetables, and higher odds of infection associated with higher intakes of processed meat [5]. Some studies have explored how intakes (mostly through interventions involving vitamins and minerals such as vitamins A, C, and D or zinc) relate to other respiratory tract infections, including the common cold or pneumonia. These studies displayed discordant results, some suggesting an impact on the incidence of infection and/or the duration of symptoms while others showed no effect [6–13]. Studies in the context of COVID-19 have mostly been ecological [14], or conducted on patients in already infected patients, focusing on the prognosis of the COVID-19 disease, in a context of tertiary prevention [15–19]. These studies provide crucial insights for disease management but they are not appropriate to investigate the potential influence of dietary habits on the risk of being infected by SARS-CoV-2, in a context of primary prevention. While individuals are continuously exposed to various pathogens (among which respiratory viruses), some are more susceptible than others to be effectively infected. Usual diet has been suggested to impact immunity in multiple ways [20] but its role on the susceptibility to SARS-CoV-2 infection is still unknown. Such information, once validated, could be useful to establish dietary guidelines aiming to lower the risk of infection by SARS-CoV-2 or other respiratory pathogens.

Our objective was therefore to study the associations between dietary intakes (nutrients, food groups, and overall diet quality) and the risk of SARS-CoV-2 infection, objectively assessed through a standardized protocol measuring the seroprevalence of anti-SARS-CoV-2 antibodies, in a large population-based sample.

## Methods

### Study population

NutriNet-Santé is a web-based cohort focusing on the relationships between nutrition and health along with the determinants of nutrition-related behaviors [21]. The recruitment of French adults started in 2009 and is still ongoing. The NutriNet-Santé study is conducted in accordance with the Declaration of Helsinki, and all procedures were approved by the Institutional Review Board of the French Institute for Health and Medical Research (IRB INSERM #0000388FWA00005831) and by the National Commission on Informatics and Liberty (CNIL #908,450 and #909,216). All participants provided informed consent and an electronic signature. The study is registered at ClinicalTrials.gov (#NCT03335644).

### Data collection

NutriNet-Santé participants regularly complete questionnaires through a dedicated and secured online platform. Upon inclusion and then every year, a set of 5 validated questionnaires collect data related to sociodemographic and lifestyle characteristics, health status and medication use, dietary intakes, physical activity (short form of the International Physical Activity Questionnaire [IPAQ]) and anthropometrics.

Dietary intakes are assessed every 6 months, each time through 3 non-consecutive, validated 24-hour dietary records, randomly distributed over 2 weeks, including 2 weekdays and 1 weekend day [22–24]. Portion sizes are estimated using validated photographs, standard containers, or directly in g/L. The food content in energy, alcohol, and macro- and micro-nutrients are derived from the NutriNet-Santé food composition table which is continuously updated and currently comprise > 3500 items. Amounts of food consumed from composite dishes are estimated using French recipes validated by food and nutrition professionals. Dietary energy under-reporters are detected via the method proposed by Black [25]. Food consumption and nutrient intakes were calculated as an average per day overall 24-h dietary records available (minimum: 6) from January 1, 2018, to February 1, 2020, i.e., the 2 years preceding the start of the COVID-19 pandemic in France.

A specific COVID-19 research protocol was set up in April 2020 as part of the SAPRIS nationwide multi-cohort project [26], including several questionnaires repeatedly collecting information about participants’ COVID-19 infection/diagnosis and experience of lockdown (e.g., employment status, presence of children at home, frequency of going out in the past week and related protective behaviors, body weight before the lockdown). All NutriNet-Santé questionnaires are available online (in French): https://info.etude-nutrinet-sante.fr/node/11.

### Assessment of SARS-CoV-2 seroprevalence

To estimate the seroprevalence of SARS-CoV-2 infection at the population level, participants who completed the SAPRIS questionnaires were invited to take part in the SAPRIS-SERO project (approved by CPP Sud-Méditerranée III on April 27, 2020, and CNIL #920193, electronic informed consent was obtained from all participants for dried-blood spot testing) [27]. Volunteer participants received self-sampling dried-blood spot kits by mail between May and October 2020. After processing, serological analyses were performed using commercial Enzyme-linked immunosorbent assay (ELISA) tests (Euroimmun®, Lübeck, Germany) to detect anti-SARS-CoV-2 antibodies (immunoglobulin G, IgG) directed against the spike protein S1 domain (ELISA-S). The ELISA-S test was considered positive for values of optical density ratio ≥1.1, indeterminate for values between 0.8 and 1.1, and negative for values < 0.8. The main outcome was a positive ELISA-S test. Participants with ELISA-S results in the indeterminate range were excluded from the analyses.

### Statistical analyses

In all, 7766 participants who provided at least 6 valid 24 h dietary records in the 2 years preceding the start of the COVID-19 pandemic in France (i.e., before February 2020) and with either a positive or a negative ELISA-S test were included in the analyses (Flowchart in Additional File 1: Fig. S1). Baseline characteristics of participants according to their seroprevalence status were described.

Associations between dietary intakes before the COVID-19 pandemic and the probability of SARS-CoV-2 infection (positive vs. negative ELISA-S test) were assessed using multivariable logistic regression models. A large range of nutritional exposures were considered to reflect the diversity of dietary intakes: from intakes of energy, macronutrients, dietary fiber, vitamins, and minerals to intakes of main food groups and indicators of the overall diet quality: the proportion of ultra-processed foods in the diet (% of food weight, assessed using the NOVA classification, as previously described [28]), the Alternative Healthy Eating Index (AHEI)-2010 score (range: 0 to 100; details in Additional File 1: Methods )[29] reflecting the adherence to a healthy diet [30], and the simplified *Programme National Nutrition Santé*-guidelines score 2 (sPNNS-GS2, range: -17 to 14.25; details in Additional file 1: Methods) [31] reflecting the adherence to the 2017 French dietary guidelines. Dietary intakes were handled as continuous variables and the odds ratios (OR) and 95% confidence intervals (CI) were computed for an increment of 1 standard deviation (SD). The log-linearity assumption was verified using restricted cubic splines [32] (no evidence of non-linearity). Models were adjusted for the following characteristics, assessed in April 2020 (i.e., during the first lockdown/wave of the epidemics in France): sex, age, educational level, employment status, smoking status, presence of children aged under 18 years at home, residential area, geographical area, frequency of going out over the past week and prevalent chronic disease (cancer, cardiovascular disease, high blood pressure, diabetes, dyslipidemia). The models also included the body mass index (BMI) and physical activity level prior to the March 2020 lockdown, as well as the month of blood draw, the number of 24 h dietary records and the intakes of energy (without alcohol) and alcohol. Finally, models included a composite index reflecting the adherence to 3 recommended protective behaviors when going out, assessed twice (questionnaires in April and May 2020). The index was calculated as the average sum of points attributed to hand washing when going back home (always-3, almost always-2, sometimes-1, never-0), mask-wearing (always-3, sometimes-1.5, never-0), and physical distancing (> 1 meter from others-3, > 1 meter from almost everybody-1.5, < 1 meter-0) and ranged from 0 to 9.

In secondary analyses, we performed multi-adjusted multinomial logistic regressions to identify dietary factors associated with seropositivity with no prior SARS-CoV-2 symptoms, or with seropositivity in participants having had SARS-CoV-2 symptoms (defined as having had at least one of the 4 following symptoms: unusual fever, cough, dyspnea, or anosmia/ageusia), compared with seronegativity. Sensitivity analyses were carried out by additionally adjusting models for the overall quality of the diet, using the sPNNS-GS2 score. Interactions were tested between each nutritional factor and sex by introducing the product of the corresponding 2 variables in the model. We also tested restricting our study sample to a nested case-control design, with matching for age, sex, and residential area (4 controls per case).

All tests were two-sided and *P*< 0.05 was considered statistically significant. Analyses were carried out using SAS 9.4 (SAS Institute Inc., USA).

## Results

Our analyses included 311 ELISA-S positive and 7455 ELISA-S negative participants (70.3% women, mean (SD) age: 60.3 years (12.9), mean number of dietary records: 10.2 (2.7)). Participants’ characteristics according to their ELISA-S status are shown in Table 1. ELISA-S positive participants were younger, more likely to have a graduate degree and a higher income, to have a professional activity during the lockdown (March–May 2020), to have children aged under 18 years at home, to have a lower level of physical activity pre-lockdown, to be non-smokers and to live in cities, and less likely to have a prevalent chronic disease. They were also more likely to have had symptoms of a SARS-CoV-2 infection, and to have been hospitalized or to have called the paramedics for SARS-CoV-2 infection symptoms. Dietary intakes according to ELISA-S status (unadjusted) are shown in Table 2.

**Table 1:** Characteristics of participants according to ELISA-S test status, NutriNet-Santé cohort study (2009–2020)—SAPRIS-SERO project

	ELISA-S negative (*n*=7455)	ELISA-S positive (*n*=311)	
	*n* (%) mean ± SD	*n* (%) mean ± SD	P*
Sex			0.19
Men	2224 (29.8)	82 (26.4)	
Women	5231 (70.2)	229 (73.6)	
Age (years)	60.6 ± 12.8	53.0 ± 13.5	< 0.0001
BMI (kg/m^2^)	23.6 ± 4.0	23.4 ± 3.8	0.24
Educational level			< 0.0001
< High-school degree	1201 (16.1)	22 (7.1)	
High-school degree	878 (11.8)	28 (9.0)	
Undergraduate degree	2361 (31.7)	92 (29.6)	
Graduate degree	3015 (40.4)	169 (54.3)	
Monthly income (household)			< 0.0001
< 1800 euros	756 (10.1)	21 (6.8)	
≥ 1800 to < 2500 euros	1124 (15.1)	34 (10.9)	
≥ 2500 to < 4000 euros	2616 (35.1)	85 (27.3)	
≥ 4000 to < 6000 euros	1833 (24.6)	101 (32.5)	
≥ 6000 euros	672 (9.0)	52 (16.7)	
Do not wish to answer	454 (6.1)	18 (5.8)	
Professional activity			< 0.0001
No professional activity prior to lockdown†	4477 (60.1)	107 (34.4)	
Short-time working‡	512 (6.9)	44 (14.2)	
Working outside home	552 (7.4)	28 (9.0)	
Working from home	1722 (23.1)	125 (40.2)	
Student, trainee	192 (2.6)	7 (2.3)	
Physical activity level pre-lockdown			0.0001
Low	1043 (14.0)	47 (15.1)	
Moderate	2931 (39.3)	156 (50.2)	
High	3481 (46.7)	108 (34.7)	
Smoking status			0.0004
Non-smoker	3161 (42.4)	165 (53.1)	
Former smoker	3900 (52.3)	138 (44.4)	
Smoker	394 (5.3)	8 (2.6)	
Residential area			< 0.0001
Rural area	2693 (36.1)	72 (23.2)	
City, < 20,000 inhabitants	1801 (24.2)	85 (27.3)	
City, ≥ 20,000 to < 100,000 inhabitants	1623 (21.8)	81 (26.1)	
City, > 100,000 inhabitants	1338 (18.0)	73 (23.5)	
Frequency of going out over the past week			0.0001
Never	533 (7.2)	44 (14.2)	
Once	1570 (21.1)	69 (22.2)	
2 to 5 times	3137 (42.1)	124 (39.9)	
6 to 10 times	1750 (23.5)	59 (19.0)	
> 10 times	465 (6.2)	15 (4.8)	
Presence of children aged under 18 years at home, yes	1139 (15.3)	110 (35.4)	< 0.0001
Prevalent cancer, yes	455 (6.1)	9 (2.9)	0.02
Prevalent cardiovascular disease or stroke, yes	354 (4.8)	9 (2.9)	0.13
Prevalent high blood pressure, yes	1519 (20.4)	50 (16.1)	0.06
Prevalent diabetes, yes	276 (3.7)	8 (2.6)	0.3
Prevalent dyslipidemia, yes	829 (11.1)	22 (7.1)	0.03
Prevalent asthma, yes	339 (4.6)	22 (7.1)	0.04
Prevalent chronic disease§, yes	2594 (34.8)	81 (26.1)	0.001
Protective behavior index||	6.1 ± 1.2	6.1 ± 1.3	0.67
Presence of COVID-19 symptoms**, yes	1541 (20.7)	166 (53.4)	< 0.0001
Admitted to hospital or called the paramedics for having COVID-19 symptoms, yes	35 (0.4)	13 (4.1)	< 0.0001
COVID-19 individual symptoms			
Fever, yes	866 (11.6)	137 (44.0)	< 0.0001
Cough, yes	1024 (13.7)	101 (32.5)	< 0.0001
Dyspnea, yes	324 (4.3)	47 (15.1)	< 0.0001
Ageusia/anosmia, yes	151 (2.0)	75 (24.1)	< 0.0001

*P values from chi-square tests (categorical variables) or Fisher tests (quantitative variables) for unadjusted associations between individual characteristics and ELISA-S test status

†Unemployed, retired, or homemaker

‡Unemployment during the COVID-19 pandemic with partial salary

§Any prevalent diseases among the following: cancer, cardio or cerebrovascular diseases, diabetes, high blood pressure, dyslipidemia, asthma

||Composite index ranging from 0 to 9 reflecting the adherence to 3 recommended protective behaviors when going out, assessed twice (questionnaires in April and May 2020). The index was calculated as the average sum of points attributed to hand washing when going back home (always-3, almost always-2, sometimes-1, never-0), mask wearing (always-3, sometimes-1.5, never-0) and physical distancing (> 1 meter from others-3, > 1 meter from almost everybody-1.5, < 1 meter-0)

**Symptoms including at least one of the following: unusual fever, cough, dyspnea, or anosmia/ageusia

**Table 2:** Dietary intakes of participants according to ELISA-S test status, NutriNet-Santé cohort study (2009–2020)—SAPRIS-SERO project

	ELISA-S negative (*n*=7455)	ELISA-S positive (*n*=311)	
	Mean ± SD	Mean ± SD	P*
**Macronutrients**			
Energy, without alcohol (kcal/day)	1800 ± 400	1800 ± 400	0.51
Alcohol (g/day)	8.5 ± 10	7.6 ± 10	0.35
Protein (g/day)	75 ± 20	74 ± 20	0.46
Carbohydrates, total (g/day)	190 ± 50	200 ± 50	0.34
Sugars (g/day)	89 ± 30	90 ± 30	0.78
Fiber (g/day)	22 ± 7	21 ± 6	0.02
Fiber, soluble (g/day)	8 ± 3	7.5 ± 2	0.004
Fiber, insoluble (g/day)	14 ± 5	13 ± 4	0.046
Fatty acids, total (g/day)	84 ± 20	85 ± 20	0.54
Saturated fatty acids (g/day)	35 ± 10	36 ± 10	0.14
Monounsaturated fatty acids (g/day)	31 ± 9	31 ± 8	0.80
Polyunsaturated fatty acids (g/day)	12 ± 4	12 ± 4	0.37
n-3 polyunsaturated fatty acids, total (g/day)	1.5 ± 0.9	1.4 ± 0.8	0.0004
n-3 linolenic acid (g/day)	1.1 ± 0.8	1.0 ± 0.7	0.006
n-3 eicosapentaenoic acid (g/day)	0.14 ± 0.1	0.11 ± 0.1	0.002
n-3 docosahexaenoic acid (g/day)	0.19 ± 0.1	0.15 ± 0.1	0.0003
n-6 polyunsaturated fatty acids, total (g/day)	10 ± 4	9.9 ± 4	0.65
Cholesterol (mg/day)	320 ± 100	308 ± 100	0.19
**Vitamins**			
Vitamin A† (μg/day)	1200 ± 600	1100 ± 500	0.03
Vitamin B1 (mg/day)	1.1 ± 0.4	1.2 ± 0.4	0.24
Vitamin B2 (mg/day)	1.7 ± 0.5	1.7 ± 0.4	0.59
Vitamin B3/PP (mg/day)	18 ± 6	18 ± 6	0.94
Vitamin B5 (mg/day)	5.2 ± 1	5.1 ± 1	0.62
Vitamin B6 (mg/day)	1.7 ± 0.5	1.7 ± 0.4	0.13
Vitamin B9 (μg/day)	330 ± 90	320 ± 90	0.004
Vitamin B12 (μg/day)	5.1 ± 4	4.5 ± 3	0.01
Vitamin C (mg/day)	110 ± 50	102 ± 40	0.002
Vitamin D (μg/day)	2.7 ± 2	2.5 ± 2	0.04
Vitamin E (mg/day)	12.0 ± 4	12 ± 4	0.41
Vitamin K (μg/day)	160 ± 100	140 ± 90	0.0001
Vitamin K from plant origin (μg/day)	130 ± 100	110 ± 80	0.0001
Vitamin K from animal origin (μg/day)	26 ± 20	25 ± 20	0.99
**Minerals**			
Selenium (μg/day)	69 ± 20	69 ± 21	0.64
Zinc (mg/day)	11 ± 3	11 ± 3	0.96
Calcium (mg/day)	940 ± 300	960 ± 300	0.15
Magnesium (mg/day)	380 ± 100	370 ± 100	0.39
Phosphorus (mg/day)	1300 ± 400	1300 ± 300	0.39
Potassium (mg/day)	3060 ± 800	2900 ± 700	0.01
Sodium (mg/day)	3060 ± 900	3007 ± 800	0.40
Copper (mg/day)	1.9 ± 0.9	1.8 ± 0.8	0.42
Iron (mg/day)	14 ± 5	14 ± 4	0.18
Iodine (μg/day)	190 ± 100	190 ± 100	0.48
Manganese (mg/day)	4.7 ± 2	4.6 ± 2	0.40
**Food groups**			
Fruit and vegetables (g/day)	460 ± 200	410 ± 200	0.0001
Legumes (g/day)	18 ± 20	17 ± 20	0.99
Nuts, unsalted (g/day)	6.9 ± 10	6.5 ± 10	0.10
Starchy foods‡ (g/day)	180 ± 90	180 ± 80	0.14
Red meat (g/day)	32 ± 30	29 ± 20	0.09
Processed meat (g/day)	27 ± 20	28 ± 30	0.87
Poultry (g/day)	20 ± 19	21 ± 20	0.28
Eggs (g/day)	17 ± 20	15 ± 10	0.01
Fish (g/day)	28 ± 20	24 ± 20	0.002
Seafood (g/day)	7.5 ± 10	6.5 ± 10	0.07
Dairy products (g/day)	180 ± 100	190 ± 100	0.03
Cakes, cookies and pastries (g/day)	48 ± 40	54 ± 40	0.02
Sweet products§ (g/day)	72 ± 50	75 ± 50	0.09
Breakfast cereals (g/day)	5.5 ± 10	7.2 ± 20	0.02
Unsweetened drinks (g/day)	1200 ± 500	1300 ± 500	0.07
Sugary drinks (g/day)	59 ± 80	64 ± 90	0.14
Alcoholic drinks (g/day)	101 ± 100	94 ± 100	0.43
Added fat, plant origin (e.g., oil) (g/day)	14 ± 8	13 ± 7	0.04
Added fat, animal origin (e.g., butter) (g/day)	9.6 ± 7	9.7 ± 8	0.72
**Overall diet quality**			
sPNNS-GS2||	2.5 ± 3	2.4 ± 3.1	0.47
AHEI-2010¶	54 ± 10	52.4 ± 11.8	0.005
Ultra-processed foods** (%)	15 ± 7	15.0 ± 6.7	0.23

*P values from Wilcoxon Mann-Whitney tests for unadjusted associations between individual characteristics and ELISA-S test status

†Total vitamin A including retinol and beta-carotene, calculated as retinol equivalent (1 mg retinol = 6 mg beta-carotene)

‡Bread, pasta, rice, potatoes, starchy vegetables, etc.

§Chocolate, sweets, honey, sugary desserts, etc.

|| *Programme National Nutrition Santé*-guidelines score 2 (simplified, sPNNS-GS2) summarizing the 2017 French dietary guidelines, ranging from ranging from − 17 to 14.25 [31]

¶Alternative Healthy Eating Index (AHEI)-2010 score, ranging from 0 to 100 [30]

**Proportion of ultra-processed foods in the diet, based on the NOVA classification [33]

**Fig. 1:**
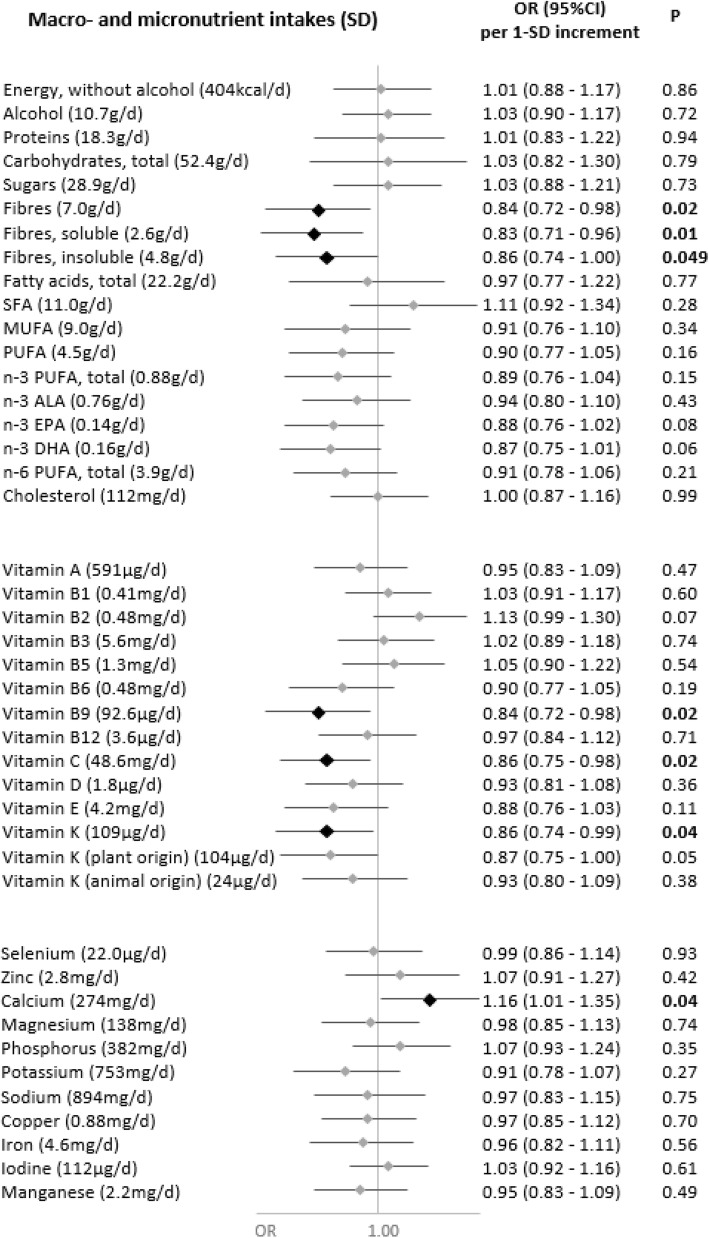
Nutritional intakes and SARS-CoV-2 infection (ELISA-S), NutriNet-Santé cohort (2009–2020)—SAPRIS-SERO. ELISA-S positive (*n*=311) compared to ELISA-S negative (*n*=7455) participants. Odds ratios and 95% confidence intervals per 1-SD increment obtained from multi-adjusted logistic regression models including sex (men/women), age, educational level (< high-school degree/high-school degree/undergraduate degree/graduate degree), employment status (no professional activity prior to lockdown: unemployed, retired, homemaker/short-time working/working outside home/working from home/student, trainee and other), smoking status (non-smoker, former smoker, smoker), presence of children and/or grandchildren aged under 18 years at home (yes/no), residential area (rural area/city < 20,000 inhabitants/city ≥ 20,000 to 100,000 inhabitants/city > 100,000 inhabitants), frequency of going out over the past week (never/once/2 to 5 times/6 to 10 times/> 10 times), prevalent chronic disease (cancer, cardiovascular disease, high blood pressure, diabetes, dyslipidemia; yes/no), geographical area (Paris Basin/Centre-East/East/Mediterranean/North/West/Paris region/Southwest), BMI and physical activity level (high, moderate, low) prior to the March 2020 lockdown, month of blood draw (May–June/July/August-September–October), number of 24 h dietary records, energy intakes (without alcohol, kcal/day; except for energy), alcohol intakes (g/day; except for alcohol) and a composite score reflecting the adherence to recommended protective behaviors when going out. Total vitamin A including retinol and beta-carotene, calculated as retinol equivalent (1 mg retinol = 6 mg beta-carotene)

**Fig. 2:**
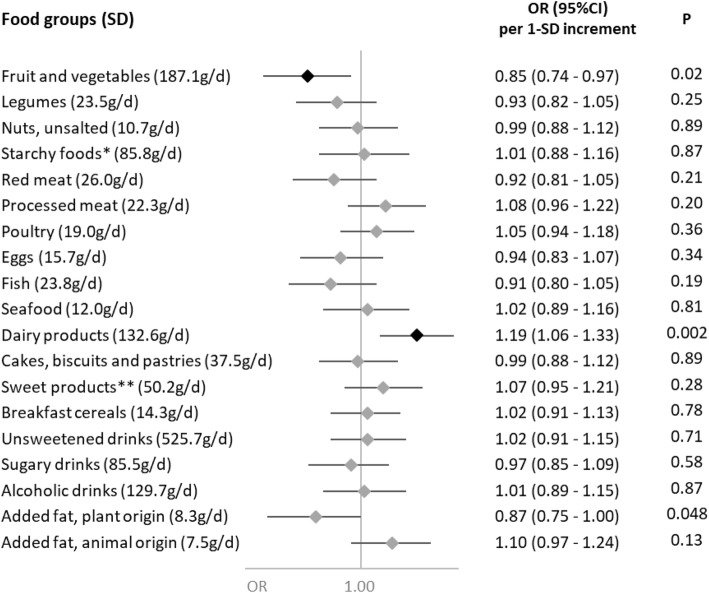
Food group consumption and SARS-CoV-2 infection (ELISA-S), NutriNet-Santé cohort (2009–2020)—SAPRIS-SERO. ELISA-S positive (*n*=311) compared to ELISA-S negative (*n*=7455) participants. Odds ratios and 95% confidence intervals per 1-SD increment obtained from multi-adjusted logistic regression models including sex (men/women), age, educational level (< high-school degree/high-school degree/undergraduate degree/graduate degree), employment status (no professional activity prior to lockdown: unemployed, retired, homemaker/short-time working/working outside home/working from home/student, trainee and other), smoking status (non-smoker, former smoker, smoker), presence of children and/or grandchildren aged under 18 years at home (yes/no), residential area (rural area/city < 20,000 inhabitants/city ≥ 20,000 to 100,000 inhabitants/city > 100,000 inhabitants), frequency of going out over the past week (never/once/2 to 5 times/6 to 10 times/> 10 times), prevalent chronic disease (cancer, cardiovascular disease, high blood pressure, diabetes, dyslipidemia; yes/no), geographical area (Paris Basin/Centre-East/East/Mediterranean/North/West/Paris region/Southwest), BMI and physical activity level (high, moderate, low) prior to the March 2020 lockdown, month of blood draw (May–June/July/August-September–October), number of 24 h dietary records, energy intakes (without alcohol, kcal/day), alcohol intakes (g/day; except for alcoholic drinks) and a composite score reflecting the adherence to recommended protective behaviors when going out. Starchy foods: bread, pasta, rice, potatoes, starchy vegetables, etc.; sugary products: chocolate, sweets, honey, sugary desserts, etc.

Associations of nutrient intakes and food consumption with the odds of SARS-CoV-2 infection are shown in Figs. 1 and 2, respectively.

Dietary intakes of vitamin B9 (OR=0.84 (0.72,0.98), *P*=0.02), vitamin C (OR=0.86 (0.75,0.98), *P*=0.02), vitamin K (OR=0.86 (0.74,0.99), *P*=0.04) (and more specifically vitamin K of plant origin (OR=0.87 (0.75,1.00), *P*=0.05)) and dietary fibers (total: OR=0.84 (0.72,0.98), *P*=0.02; soluble: OR=0.83 (0.71,0.96), *P*=0.01; insoluble: OR=0.86 (0.74,1.00), *P*=0.049), and, consistently, the consumption of fruits and vegetables (OR=0.85 (0.74,0.97), *P*=0.02), were associated with decreased odds of SARS-CoV-2 infection.

Conversely, higher intakes of calcium (OR=1.16 (1.01,1.35), *P*=0.04) and dairy products (OR=1.19 (1.06,1.33), *P*=0.002) were associated with increased odds of SARS-CoV-2 infection. This association was especially observed for milk (OR=1.15 (1.03,1.27), *P*=0.01), with a similar trend for yogurt (OR=1.12 (1.00,1.25), *P*=0.06), but not for cheese (OR=0.96 (0.84,1.09), *P*=0.54) or cottage cheese (OR=1.03 (0.92,1.16), *P*=0.60).

The food groups that contributed the most to the intakes of these nutrients were fruits and vegetables and starchy foods for fibers and vitamin B9, fruits and vegetables and sugary drinks for vitamin C, fruits and vegetables, starchy foods, and dairy products for vitamin K, and dairy products for calcium (Additional file 1: Fig. S2).

No association was observed neither with the other nutrients or food groups nor with the overall dietary scores AHEI-2010 (OR=0.96 (0.85,1.09), *P*=0.52), sPNNS-GS2 (OR=0.95 (0.82,1.10), *P*=0.45), or with the proportion of ultra-processed food in the diet (OR=0.96 (0.85,1.08), *P*=0.52).

Sensitivity analyses with additional adjustment for the overall dietary score sPNNS-GS2 provided similar results (Additional file 1: Table S1).

Only two interactions were detected with sex. While soluble fiber intake exhibited the same trend but only in women (P-interaction=0.02; women: OR=0.70 (0.58,0.85), *P*=0.0003; men: OR=1.12 (0.87,1.44), *P*=0.37), a significant association with the risk of SARS-CoV-2 infection was detected in women for the intake of sweet products (P-interaction=0.01; women: OR=1.17 (1.02,1.35), *P*=0.03; men: OR=0.83 (0.64,1.07), *P*=0.14).

**Fig. 3:**
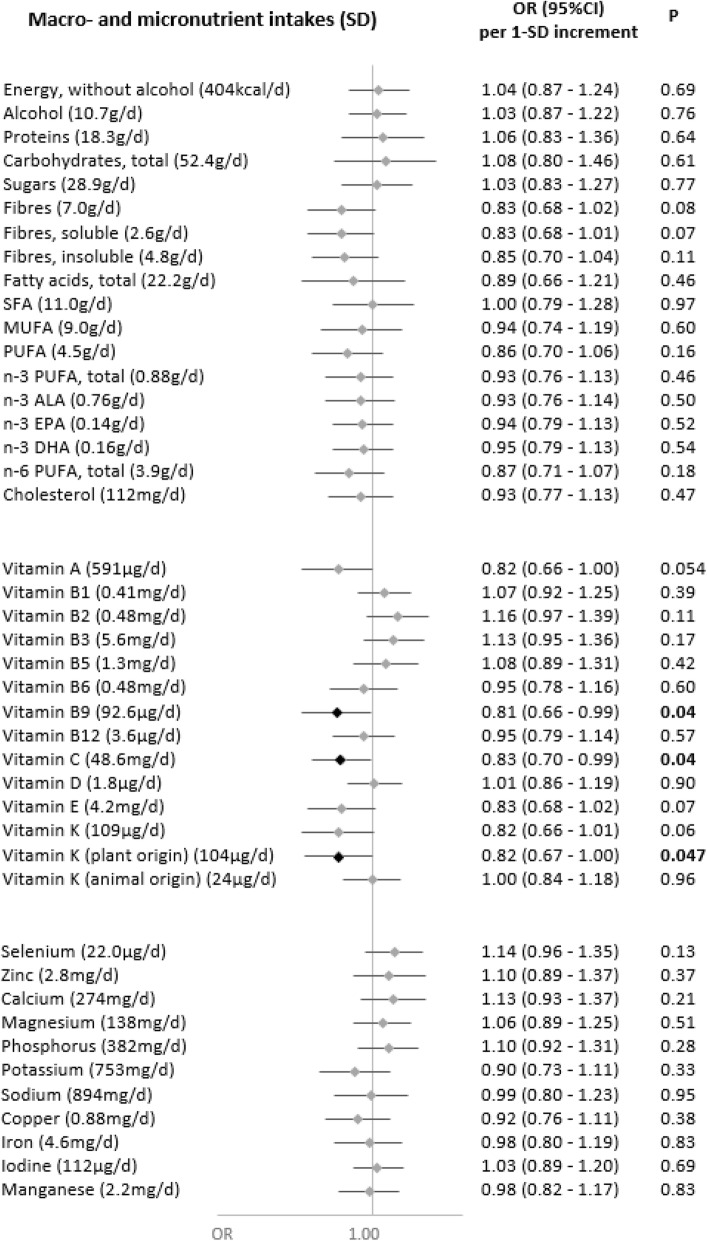
Nutritional intakes and symptomatic SARS-CoV-2 infection (ELISA-S), NutriNet-Santé cohort (2009–2020)—SAPRIS-SERO. Symptomatic ELISA-S positive (*n*=179) compared to ELISA-S negative (*n*=7455) participants. Odds ratios and 95% confidence intervals per 1-SD increment obtained from multi-adjusted logistic regression models including sex (men/women), age, educational level (< high-school degree/high-school degree/undergraduate degree/graduate degree), employment status (no professional activity prior to lockdown: unemployed, retired, homemaker/short-time working/working outside home/working from home/student, trainee and other), smoking status (non-smoker, former smoker, smoker), presence of children and/or grandchildren aged under 18 years at home (yes/no), residential area (rural area/city < 20,000 inhabitants/city ≥ 20,000 to 100,000 inhabitants/city > 100,000 inhabitants), frequency of going out over the past week (never/once/2 to 5 times/6 to 10 times/> 10 times), prevalent chronic disease (cancer, cardiovascular disease, high blood pressure, diabetes, dyslipidemia; yes/no), geographical area (Paris Basin/Centre-East/East/Mediterranean/North/West/Paris region/Southwest), BMI and physical activity level (high, moderate, low) prior to the March 2020 lockdown, month of blood draw (May–June/July/August–September–October), number of 24 h dietary records, energy intakes (without alcohol, kcal/day; except for energy), alcohol intakes (g/day; except for alcohol) and a composite score reflecting the adherence to recommended protective behaviors when going out. Total vitamin A including retinol and beta-carotene, calculated as retinol equivalent (1 mg retinol = 6 mg beta-carotene)

**Fig. 4:**
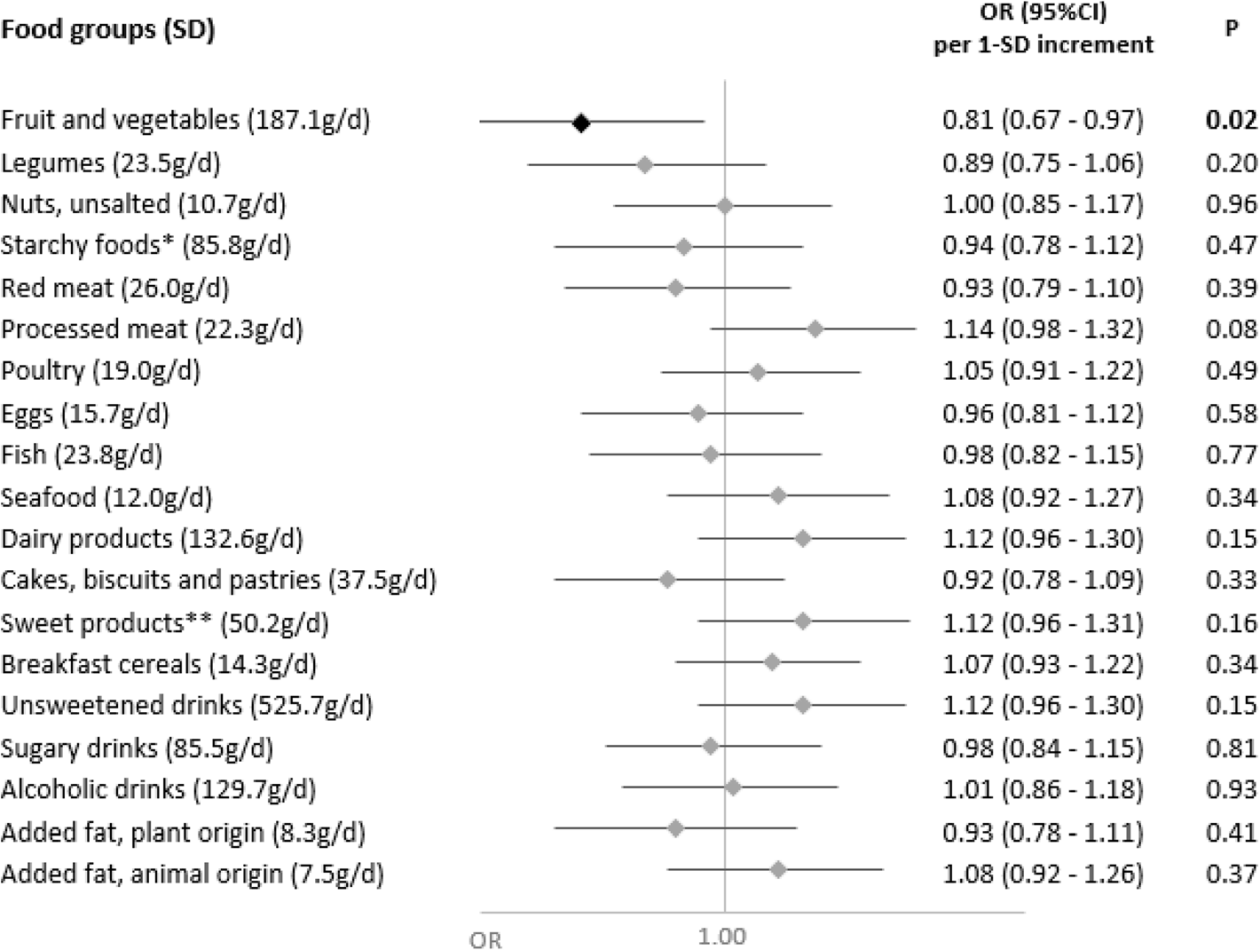
Food group consumption and symptomatic SARS-CoV-2 infection (ELISA-S), NutriNet-Santé cohort (2009–2020)—SAPRIS-SERO. Symptomatic ELISA-S positive (*n*=179) compared to ELISA-S negative (*n*=7455) participants. Odds ratios and 95% confidence intervals per 1-SD increment obtained from multi-adjusted logistic regression models including sex (men/women), age, educational level (< high-school degree/high-school degree/undergraduate degree/graduate degree), employment status (no professional activity prior to lockdown: unemployed, retired, homemaker/short-time working/working outside home/working from home/student, trainee and other), smoking status (non-smoker, former smoker, smoker), presence of children and/or grandchildren aged under 18 years at home (yes/no), residential area (rural area/city < 20,000 inhabitants/city ≥ 20,000 to 100,000 inhabitants/city > 100,000 inhabitants), frequency of going out over the past week (never/once/2 to 5 times/6 to 10 times/> 10 times), prevalent chronic disease (cancer, cardiovascular disease, high blood pressure, diabetes, dyslipidemia; yes/no), geographical area (Paris Basin/Centre-East/East/Mediterranean/North/West/Paris region/Southwest), BMI and physical activity level (high, moderate, low) prior to the March 2020 lockdown, month of blood draw (May–June/July/August–September–October), number of 24 h dietary records, energy intakes (without alcohol, kcal/day), alcohol intakes (g/day; except for alcoholic drinks) and a composite score reflecting the adherence to recommended protective behaviors when going out. Starchy foods: bread, pasta, rice, potatoes, starchy vegetables, etc.; sugary products: chocolate, sweets, honey, sugary desserts, etc.

**Fig. 5:**
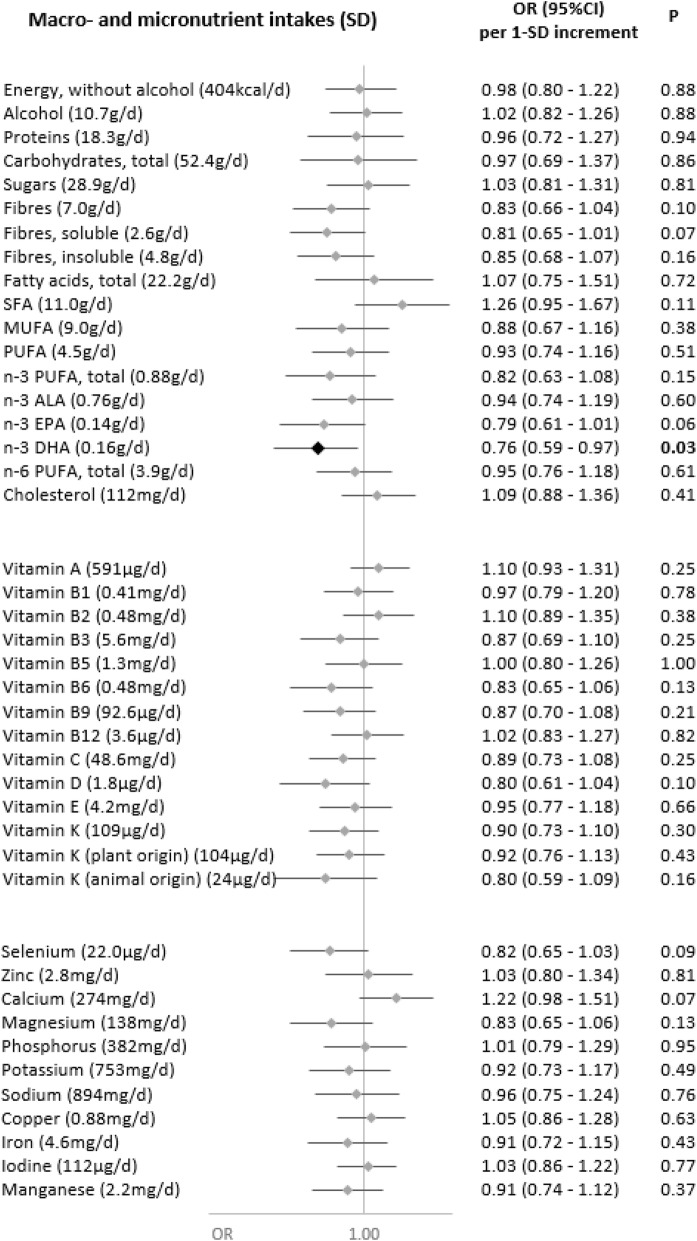
Nutritional intakes and asymptomatic SARS-CoV-2 infection (ELISA-S), NutriNet-Santé cohort (2009–2020)—SAPRIS-SERO. Asymptomatic ELISA-S positive (*n*=132) compared to ELISA-S negative (*n*=7455) participants. Odds ratios and 95% confidence intervals per 1-SD increment obtained from multi-adjusted logistic regression models including sex (men/women), age, educational level (< high-school degree/high-school degree/undergraduate degree/graduate degree), employment status (no professional activity prior to lockdown: unemployed, retired, homemaker/short-time working/working outside home/working from home/student, trainee and other), smoking status (non-smoker, former smoker, smoker), presence of children and/or grandchildren aged under 18 years at home (yes/no), residential area (rural area/city < 20,000 inhabitants/city ≥ 20,000 to 100,000 inhabitants/city > 100,000 inhabitants), frequency of going out over the past week (never/once/2 to 5 times/6 to 10 times/> 10 times), prevalent chronic disease (cancer, cardiovascular disease, high blood pressure, diabetes, dyslipidemia; yes/no), geographical area (Paris Basin/Centre-East/East/Mediterranean/North/West/Paris region/Southwest), BMI and physical activity level (high, moderate, low) prior to the March 2020 lockdown, month of blood draw (May–June/July/August–September–October), number of 24 h dietary records, energy intakes (without alcohol, kcal/day; except for energy), alcohol intakes (g/day; except for alcohol) and a composite score reflecting the adherence to recommended protective behaviors when going out. Total vitamin A including retinol and beta-carotene, calculated as retinol equivalent (1 mg retinol = 6 mg beta-carotene)

**Fig. 6:**
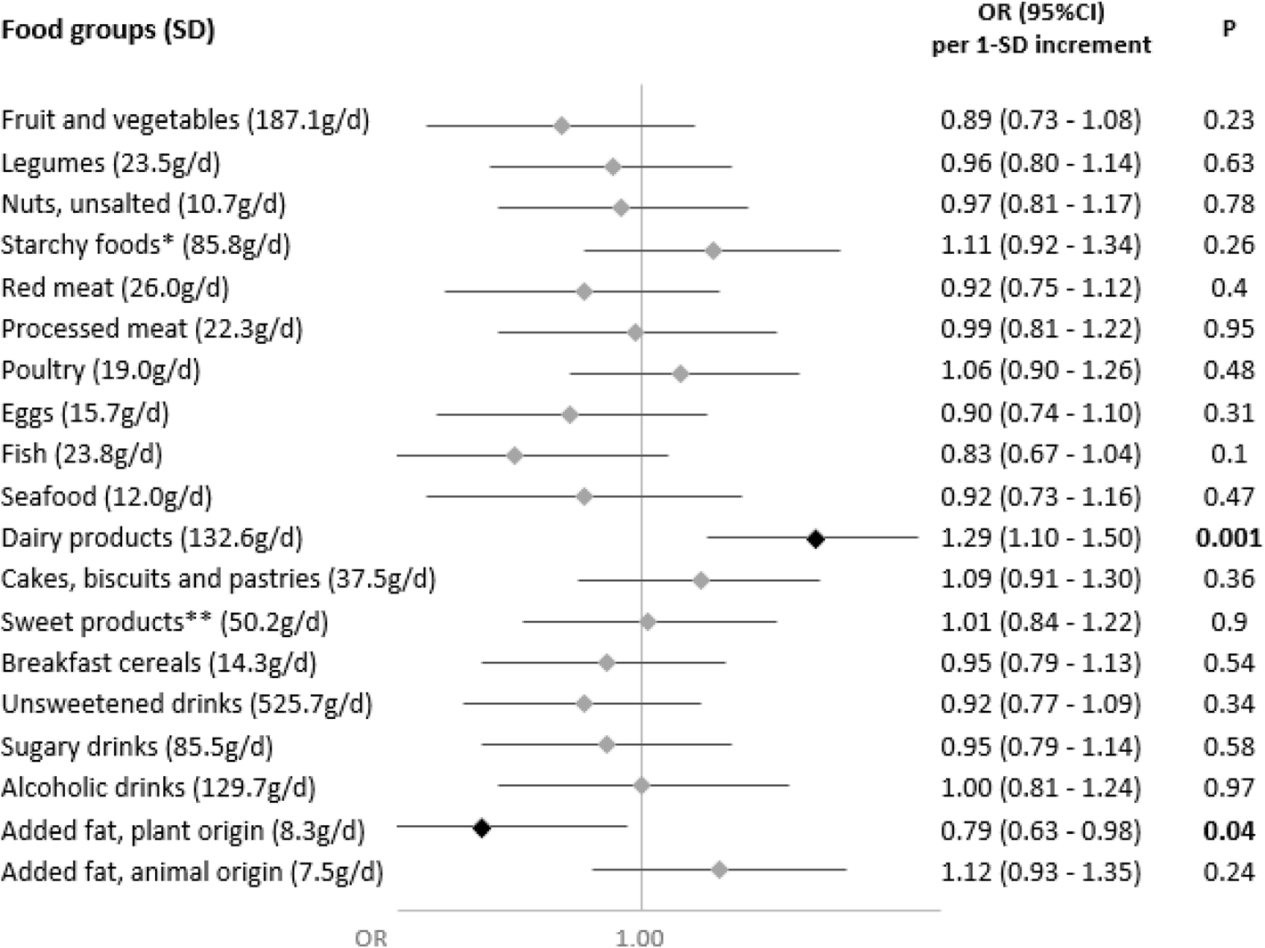
Food group consumption and asymptomatic SARS-CoV-2 infection (ELISA-S), NutriNet-Santé cohort (2009–2020)—SAPRIS-SERO. Asymptomatic ELISA-S positive (*n*=132) compared to ELISA-S negative (*n*=7455) participants. Odds ratios and 95% confidence intervals per 1-SD increment obtained from multi-adjusted logistic regression models including sex (men/women), age, educational level (< high-school degree/high-school degree/undergraduate degree/graduate degree), employment status (no professional activity prior to lockdown: unemployed, retired, homemaker/short-time working/working outside home/working from home/student, trainee and other), smoking status (non-smoker, former smoker, smoker), presence of children and/or grandchildren aged under 18 years at home (yes/no), residential area (rural area/city < 20,000 inhabitants/city ≥ 20,000 to 100,000 inhabitants/city > 100,000 inhabitants), frequency of going out over the past week (never/once/2 to 5 times/6 to 10 times/> 10 times), prevalent chronic disease (cancer, cardiovascular disease, high blood pressure, diabetes, dyslipidemia; yes/no), geographical area (Paris Basin/Centre-East/East/Mediterranean/North/West/Paris region/Southwest), BMI and physical activity level (high, moderate, low) prior to the March 2020 lockdown, month of blood draw (May–June/July/August–September–October), number of 24 h dietary records, energy intakes (without alcohol, kcal/day), alcohol intakes (g/day; except for alcoholic drinks) and a composite score reflecting the adherence to recommended protective behaviors when going out. Starchy foods: bread, pasta, rice, potatoes, starchy vegetables, etc.; sugary products: chocolate, sweets, honey, sugary desserts, etc.

In secondary analyses, we explored the associations of nutrient intakes and food groups with the odds of having a symptomatic or asymptomatic SARS-CoV-2 infection (Figs. 3, 4, 5, and 6). Intakes of fruit and vegetables as well as vitamins C, K (from plant-based products), and B9 were inversely associated with the odds of a symptomatic SARS-CoV-2 infection. As regards asymptomatic infections, inverse associations were observed for DPA-omega 3 fatty acids and added fats of plant origin, and direct associations were observed for dairy products.

Sensitivity analyses on the nested case-control sample provided similar results (Additional file 1: Figs. S3-S4): higher intakes of fiber, vitamin B9, vitamin C, fruits, and vegetables were associated with lower odds of being seropositive for SARS-CoV-2, while direct associations were observed for dairy products, consistently with the main models. Few differences were observed: vitamin E (OR=0.84 (0.71,0.99), *P*=0.04) and added fat of plant origin (OR=0.82 (0.70,0.95), *P*=0.01) were inversely associated with the risk of infection, and direct associations were observed with added fats of animal origin (OR=1.16 (1.01,1.32), *P*=0.03). On the other hand, associations with vitamin K and calcium were slightly attenuated and became non-significant (*P*=0.07 and 0.09, respectively).

## Discussion

Our results showed that a diet rich in fruit and vegetables, consistent with higher intakes of vitamin C, vitamin B9, vitamin K, and dietary fiber was associated with a lower probability of SARS-CoV-2 infection, as reflected by seroprevalence data. We also observed that higher intakes of dairy products and calcium were associated with higher odds of SARS-CoV-2 infection. More specifically, higher intakes of vitamins C, K (from plant-based foods), and B9, as well as fruits and vegetables were associated with a lower probability of a symptomatic SARS-CoV-2 infection.

To the best of our knowledge, our study was the first prospective study to use objective serology data, coupled with detailed dietary assessment, to examine the associations between usual diet and the odds of having been infected by SARS-CoV-2. A prospective Spanish study in the SUN cohort observed an association between a better adherence to the Mediterranean diet and a lower risk of SARS-CoV-2 infection [4]. Nevertheless, the outcome assessment in this study was based on self-reported questionnaires collecting SARS-CoV-2 diagnostic tests, and not seroprevalence. On the other hand, a study in the UK-Biobank reported a protective association for higher intakes of coffee and vegetables and higher odds of infection associated with higher intakes of processed meat [5]. However, this study was conducted only among participants who sought COVID-19 testing, and based on a non-detailed dietary assessment, using a simplified 17-item questionnaire.

Vitamin C is a well-known actor of the immune system and could help prevent SARS-CoV-2 infection notably through an enhanced innate immune response with the maintenance of the epithelial barrier defending against pathogens and the promotion of phagocytosis (regulation of neutrophil function, notably increased chemotactic response) [6, 11, 34]. Although rare in developed countries, severe vitamin C deficiency (scurvy) has been observed to predispose individuals to infections, especially of the respiratory tract, like pneumonia [34]. Yet, randomized trials have mostly failed to demonstrate a beneficial effect of vitamin C regarding the common cold, upper respiratory tract infections (URTI), or pneumonia, showing only a weak inconsistent effect on the incidence of infection, but a clearer effect on the duration of symptoms [6–10, 34]. However, these trials usually did not consider dietary intakes of vitamin C, which may have limited the observation of effects if baseline nutritional status was already adequate [35]. Moreover, a prospective cohort study among Swedish adults observed a decreased risk of URTI associated with higher dietary intakes of vitamin C in women [12]. Finally, some studies were performed in the context of COVID-19 but focusing on hospitalized patients, observing low vitamin C status in critically ill patients [36], and suggesting improved outcomes with high-dose vitamin C treatment [16]. Largely consistent with the current evidence [37], our results suggest that higher vitamin C intakes as part of the regular diet may decrease the risk of SARS-CoV-2 infection, probably through an enhanced early immune response.

Vitamin B9 (folate) has also been suggested as a potential helper in preventing SARS-CoV-2 infection, through a role in innate immunity and in particular for natural killer cell cytotoxicity, which may reduce the risk of infection, as evidenced in an intervention study in older adults [3]. Evidence more specifically related to SARS-CoV-2 from computer simulation studies suggested that vitamin B9 could prevent the virus from entering the cells through an inhibition of furin, a protease involved in the activation of the spike protein, and from replicating through an inactivation of the protease 3CL^*pro*^ [18]. Thus, our results are consistent with those mostly mechanistic data; strong direct evidence of the potential of vitamin B9 to prevent infection remains limited at present.

Vitamin K is known to play a role in the regulation of blood coagulation and calcification of bone and vessel tissue. In addition, vitamin K could be involved in the regulation of innate immunity, with a stimulation of apoptotic cell clearance by phagocytes, and of inflammatory responses [38]. Hence, it has been suggested that vitamin K could provide some benefits against COVID-19 complications [17, 39]. Our data suggest that it could also help prevent the infection by SARS-CoV-2.

The potential effect of dietary fibers on the risk of SARS-CoV-2 infection is likely resulting from their interaction with the gut microbiota, producing short-chain fatty acids (SCFA) and promoting a diverse and balanced community. The importance of the gut microbiota for efficient and balanced immune and inflammatory responses is well-established. In particular, SCFA contribute to the innate immune response through enhanced production of reactive oxygen species and phagocytosis, apoptosis, and modulation of neutrophil recruitment [40]. Studies in animals have shown the importance of gut microbiota and dietary fiber intakes in the response against viral infection [41, 42]. Hence, although direct evidence in humans is scarce, our results are in line with the mechanistic evidence linking dietary fibers, the gut microbiota, and the immune function.

Consistent with our results observed for vitamins C, B9, K, and dietary fiber, we also observed a decreased susceptibility of SARS-CoV-2 infection with higher intakes of fruit and vegetables. Fruit and vegetables indeed provide a mix of these highly relevant compounds for the immune function (as discussed above), and also other bioactive compounds such as polyphenols [43]. Our findings regarding the consumption of vegetables are consistent with those of the recently published prospective study in the UK-Biobank [5]. A retrospective study in pregnant women also suggested a moderate effect of fruit and vegetable intake in the prevention of URTI [13]. In contrast, a study observed that countries with higher consumptions of fruit had higher infection and mortality rates by COVID-19 but it was based on an ecological design, which did not allow the adjustment for individual factors that may cofound the studied associations [44]. The fact that we observed associations with several compounds provided by fruit and vegetables makes it difficult to disentangle the specific effect of each compound, which will need to be explored in experimental studies and randomized trials.

Whereas most of the associations we observed were in line with mechanistic evidence and current hypotheses regarding the potential role of nutrition in the prevention of SARS-CoV-2 infection, we also observed an unexpected association between higher intakes of dairy products (especially milk) and calcium and a higher likelihood of SARS-CoV-2 infection. Indeed, dairy product intake is usually considered as beneficial for the immune system, anti-inflammatory processes, and the response to infections [45]. These findings, along with those on fruits and vegetables, are somehow consistent with a Spanish prospective study [4], reporting that lower odds of COVID-19 infection are associated with a higher adherence to Mediterranean diet (i.e., a diet with high intakes of fruits and vegetables and limited intakes of dairy products). A possible mechanism that could partly explain this increased risk involves a calcium-sensitive interaction of SARS-CoV-2 with the ACE-2 receptor (its entry point in human cells). Hence, more available calcium could ease the entry of SARS-CoV-2 in the host cells [46]. However, this result remains unclear and warrants further investigation.

Finally, other nutrients that were expected to have a potential beneficial effect against SARS-CoV-2 infection (e.g., selenium, zinc, copper, iron, vitamins A, D, and E) [6, 47] did not display associations with the risk of SARS-CoV-2 infection in our study. This could potentially relate to the nutritional status of our participants for these nutrients, with perhaps an appropriate status for immune function in most, and a relatively reduced range of intakes among participants. These nutrients could also be involved to mitigate the severity of symptoms or the prognosis once infected, which was beyond the scope of this study focusing on the susceptibility to infection. As regards vitamin D, meta-analyses have shown a potential benefit of a vitamin D supplementation for COVID-19 outcomes and mortality [48]; similarly, a meta-analysis showed a trend for a deleterious association between low serum 25(OH)D levels and COVID-19 related health outcomes [49]. However, diet is not the major contributor to vitamin D intake, compared with sun exposure and the use of dietary supplements or medication. Specific investigation of vitamin D accounting for all its sources constitutes a perspective of this research work. Likewise, we did not observe associations with the indicators of overall diet quality. This tends to suggest a specific effect of some components playing a key role in immune function and the prevention of SARS-CoV-2 infection, which may be diluted when considering these overall indicators.

Main strengths of our study include the comprehensive assessment of SARS-CoV-2 seroprevalence with highly sensitive assays in a large sample, independent of whether or not the participant sought testing (contrary to studies using PCR results retrieved from medical records) or even had symptoms (that might not be specific to SARS-CoV-2 infections), and the detailed characterization of dietary intakes of participants prior to the pandemic (at least 6 and an average of 10.2 validated 24 h dietary records) allowing us to relate habitual diet to SARS-CoV-2 infection with a prospective design. The presence of antibodies indicates that a person has been infected with SARS-CoV-2, but does not indicate the exact date of the infection. Therefore, to guarantee as much as possible a prospective design, dietary data were considered until February 2020, the start of the epidemic in France. Yet, some limitations should be acknowledged. First, studies have suggested that the ELISA test has imperfect sensitivity (85–90%) [50, 51] and that anti-SARS-CoV-2 antibodies may decrease over time, which may have resulted in misclassification in a way that a person who has been infected with the virus might have not been positive according to the ELISA test; however, the date of collection of dried-blood spot kits was between May and October 2020 (i.e. not a very long time-frame since the beginning of the pandemic in France). Furthermore, the extent to which participants have actually been exposed to the virus is difficult to assess; hence, our results might be confused by behavioral factors, such as an increase awareness and application of protective measures in people who are more health-conscious and have a more balanced diet. To mitigate this potential bias, our models included numerous covariates related to the risk of being exposed to the virus (e.g., employment status during the lockdown, presence of children at home, index reflecting the application of protective measures). In addition, some data were not available in our study. In France, the collection of racial and ethnic data is generally not permitted in the framework of cohort studies, thus, these data were not available. Moreover, quantitative data about the use of dietary supplements (relevant when investigating specific nutrients such as vitamin D) was not yet available and thus, has not been included in this study. Details about subtypes of vitamin K (K1 and K2) were not available; even though these 2 subtypes might have similar biological functions, differences pertain to bioavailability and tissue distribution [52]. However, because dietary sources of vitamin K1 and K2 are distinct (plant or animal sources, respectively), we used these as proxy. Next, the seronegative and seropositive groups differed at baseline according to several characteristics that constitute potential confounders susceptible to influence the studied associations in both directions. For instance, older age and higher physical activity levels were associated with healthier diets and lower risk of infection (which would tend to strengthen the observed associations), but the higher educational level was associated with healthier diets and higher risk of infection (which would tend to attenuate the observed associations). However, even though residual confounding (linked to unmeasured factors or inaccuracies in data assessment) cannot be totally ruled out, we largely accounted for potential confounding by adjusting all models for a wide panel of covariates (e.g., age, sex, educational, professional and socio-economical levels, smoking status, behaviors during the pandemic…) and by matching for age, sex and residential area in sensitivity analyses. Finally, as a long-term study on nutrition and health, NutriNet-Santé includes more women and individuals with overall higher socioeconomic status and healthier lifestyle and dietary habits compared to the general French population. This may limit the generalizability of our findings and might also have resulted in a smaller range in dietary intakes, thus a loss of statistical power.

## Conclusions

In this study, conducted in a large population-based sample with seroprevalence data, fruit and vegetable intakes, and, consistently, dietary intakes of vitamin C, folates, vitamin K and fiber were associated with a lower susceptibility to SARS-CoV-2 infection. Further studies are needed in order to better understand the role played by dietary habits on the risk of SARS-CoV-2 infection. Insights from different approaches should help build robust scientific evidence. This includes well-designed randomized controlled trials (for factors with protective hypotheses), other observational prospective studies with detailed information about dietary exposures (including blood markers of micronutrient status) and outcome (including information on early symptoms and severity) to capture real-life behaviors in different countries and settings, and mechanistic approaches, using metabolomics, for instance, to understand the underlying pathways. Beyond its established role in the prevention of non-communicable chronic diseases [53], nutrition could therefore provide one strategic leverage to improve the immune function at the population level and contribute to the protection against SARS-CoV-2 infection.

## Supplementary Information

**Additional file 1: Supplementary Methods.** Computation of the simplified Programme National Nutrition Santé-guidelines score 2 (sPNNS-GS2) and of the Alternative Healthy Eating Index (AHEI)-2010. **Supplementary Figure S1.** Participants flowchart, NutriNet-Santé cohort (2009-2020) – SAPRIS-SERO. **Supplementary Table S1.** Nutritional intakes, food group consumption and SARS-CoV-2 infection (ELISA-S), sensitivity analyses, NutriNet-Santé cohort (2009-2020) – SAPRIS-SERO. **Supplementary Figure S2.** Food group contributions to the intakes of nutrients, NutriNet-Santé cohort (2009-2020) – SAPRIS-SERO. **Supplementary Figure S3.** Nutritional intakes and SARS-CoV-2 infection (ELISA-S) with a nested case-control design, NutriNet-Santé cohort (2009-2020) – SAPRIS-SERO. **Supplementary Figure S4.** Food group consumption and SARS-CoV-2 infection (ELISA-S) with a nested case-control design, NutriNet-Santé cohort (2009-2020) – SAPRIS-SERO.

## Data Availability

Researchers from public institutions can submit a collaboration request including their institution and a brief description of the project to collaboration@etude-nutrinet-sante. All requests will be reviewed by the steering committee of the NutriNet-Santé study. A financial contribution may be requested. If the collaboration is accepted, a data access agreement will be necessary and appropriate authorizations from the competent administrative authorities may be needed. In accordance with existing regulations, no personal data will be accessible.

## Abbreviations

COVID-19: Coronavirus disease 2019
AHEI: Alternative Healthy Eating Index
BMI: Body mass index
CI: Confidence interval
ELISA: Enzyme-linked immunosorbent assay
IgG: Immunoglobulin G
IPAQ: International Physical Activity Questionnaire
OR: Odds ratio
PNNS-GS2: *Programme National Nutrition Santé*-guidelines score 2
RT-PCR: Real-time polymerase chain reaction tests
SARS-CoV-2: Severe acute respiratory syndrome coronavirus 2
SD: Standard deviation
URTI: Upper respiratory tract infections
