# Supplementary material for: Nutritional risk factors for SARS-CoV-2 infection: a prospective study within the NutriNet-Santé cohort
Source: BMC Med. 2021 Nov 30;19:290. doi: 10.1186/s12916-021-02168-1 (PMC8629697; doi:10.1186/s12916-021-02168-1)
Supplement: Supplementary file 1 — Additional file 1: Supplementary Methods. Computation of the simplified Programme National Nutrition Santé-guidelines score 2 (sPNNS-GS2) and of the Alternative Healthy Eating Index (AHEI)-2010. Supplementary Figure S1. Participants flowchart, NutriNet-Santé cohort (2009-2020) – SAPRIS-SERO. Supplementary Table S1. Nutritional intakes, food group consumption and SARS-CoV-2 infection (ELISA-S), sensitivity analyses, NutriNet-Santé cohort (2009-2020) – SAPRIS-SERO. Supplementary Figure S2. Food group contributions to the intakes of nutrients, NutriNet-Santé cohort (2009-2020) – SAPRIS-SERO. Supplementary Figure S3. Nutritional intakes and SARS-CoV-2 infection (ELISA-S) with a nested case-control design, NutriNet-Santé cohort (2009-2020) – SAPRIS-SERO. Supplementary Figure S4. Food group consumption and SARS-CoV-2 infection (ELISA-S) with a nested case-control design, NutriNet-Santé cohort (2009-2020) – SAPRIS-SERO. [file 12916_2021_2168_MOESM1_ESM.docx]

**ADDITIONAL FILE 1 - CONTENTS**

**Supplementary Methods.** Computation of the simplified Programme National Nutrition Santé-guidelines score 2 (sPNNS-GS2) and of the Alternative Healthy Eating Index (AHEI)-2010

**Supplementary Figure S1.** Participants flowchart, NutriNet-Santé cohort (2009-2020) – SAPRIS-SERO

**Supplementary Table S1.** Nutritional intakes, food group consumption and SARS-CoV-2 infection (ELISA-S), sensitivity analyses, NutriNet-Santé cohort (2009-2020) – SAPRIS-SERO.

**Supplementary Figure S2.** Food group contributions to the intakes of nutrients, NutriNet-Santé cohort (2009-2020) – SAPRIS-SERO

**Supplementary Figure S3.** Nutritional intakes and SARS-CoV-2 infection (ELISA-S) with a nested case-control design, NutriNet-Santé cohort (2009-2020) – SAPRIS-SERO.

**Supplementary Figure S4.** Food group consumption and SARS-CoV-2 infection (ELISA-S) with a nested case-control design, NutriNet-Santé cohort (2009-2020) – SAPRIS-SERO.

**Supplementary Methods**

**Computation of the simplified *Programme National Nutrition Santé*-guidelines score 2 (sPNNS-GS2)**

The sPNNS-GS2 score has been published previously. Scores were computed for each participant as the sum of each component multiplied by its associated weight and divided by its maximum absolute value. The theoretical range was -17 to 14.25.

| **Dietary components** | **Recommendation** | **Criteria^a^** | **Score** |
| --- | --- | --- | --- |
| Fruits and vegetables | **At least 5 servings/day, with 1 max as juice and 1 max as dried** | [0 - 3.5[ | 0 |
| (weight=3) |  | [3.5 - 5[ | 0.5 |
|  |  | [5 - 7.5[ | 1 |
|  |  | ≥7.5 | 2 |
| Nuts | **A handful/day** | 0 | 0 |
| (weight=1) |  | ]0 – 0.5[ | 0.5 |
|  |  | [0.5 - 1.5[ | 1 |
|  |  | ≥1.5 | 0 |
| Legumes | **At least 2 servings/week** | 0 /week | 0 |
| (weight=1) |  | ]0 - 2[ /week | 0.5 |
|  |  | ≥2 /week | 1 |
| Whole-grain food | **Every day** | 0 | 0 |
| (weight=2) |  | ]0 - 1[ | 0.5 |
|  |  | [1 - 2[ | 1 |
|  |  | ≥2 | 1.5 |
| Milk and dairy products | **2 servings/day** | [0 - 0.5[ | 0 |
| (weight=1) |  | [0.5 - 1.5[ | 0.5 |
|  |  | [1.5 - 2.5[ | 1 |
|  |  | ≥2.5 | 0 |
| Red meat | **Limit consumption** | [0 - 500[ g/week | 0 |
| (weight=2) |  | [500 - 750[ g/week | -1 |
|  |  | ≥750 g/week | -2 |
| Processed meat | **Limit consumption** | [0 - 150[ g/week | 0 |
| (weight=3) |  | [150 - 300[ g/week | -1 |
|  |  | ≥300 g/week | -2 |
| Fish and seafood | **2 servings/week** | [0 - 1.5[ servings/week | 0 |
| (weight=2) |  | [1.5 - 2.5[ servings/week | 1 |
|  |  | [2.5 - 3.5[ servings/week | 0.5 |
|  |  | ≥3.5 servings/week | 0 |
| Added fat | **Avoid overeating** | >16% of EIWA ^c^ | 0 |
| (weight=2) |  | ≤16% of EIWA | 1.5 |
| Sugary foods | **Limit consumption** | <10% of EIWA | 0 |
| (weight=3) |  | [10 - 15[% of EIWA | -1 |
|  |  | ≥15% of EIWA | -2 |
| Sweet-tasting beverages^b^ | **Limit consumption** | 0 mL/day | 0 |
| (weight=3) |  | ]0 - 250[ mL/day | -0.5 |
|  |  | [250 - 750[ mL/day | -1 |
|  |  | ≥750mL mL/day | -2 |
| Alcoholic beverages | **Limit consumption** | 0 g/week | 0.5 |
| (weight=3) |  | ]0 - 100] g/week | 0 |
|  |  | ]100 - 150] g/week | -1 |
|  |  | ]150 - 200] g/week | -1.5 |
|  |  | >200 g/week | -2 |
| Salt | **Limit consumption** | <6 g/day | 1 |
| (weight=3) |  | [6 - 8[ g/day | 0 |
|  |  | [8 - 10[ g/day | -0.5 |
|  |  | [10 - 12[ g/day | -1 |
|  |  | ≥12 g/day | -2 |

^a^ Servings per d unless stated otherwise.

^b^ Sugary beverages, artificially sweetened beverages and fruit juices.

**Computation of the Alternative Healthy Eating Index (AHEI)-2010**

The AHEI-2010 score has been published previously.

The computation in the NutriNet-Santé cohort included 10 out of 11 original components as the intake of trans fatty acids was not available. Each component ranges from 0 to 10. Criteria for minimum and maximum scores are shown in the table below. Intermediate scores are attributed proportionally to the intake (see an example for the vegetable component in footnote 1)

| **Component** | **Criteria for minimum score (0)** | **Criteria for maximum score (10)** |
| --- | --- | --- |
| Vegetables^1^, servings/d | 0 | ≥ 5 |
| Fruit^2^, servings/d | 0 | ≥ 4 |
| Whole grains, g/d | 0 | Women: 75  Men: 90 |
| Sugar-sweetened beverages and fruit juice^3^, servings/d | ≥ 1 | 0 |
| Nuts and legumes^4^, servings/d | 0 | ≥ 1 |
| Red or processed meat^5^, servings/d | ≥ 1.5 | 0 |
| Long-chain (n-3) PUFA (EPA + DHA), mg/d | 0 | 250 |
| PUFA, % of energy | ≤ 2 | ≥ 10 |
| Sodium^6^, mg/d | Highest decile | Lowest decile |
| Alcohol^7^, drinks/d | Women: ≥ 2.5  Men: ≥ 3.5 | Women: 0.5-1.5  Men: 0.5-2.0 |
| **Total** | **0** | **100** |

^1^ Potatoes and tubers are not included in the vegetable component; 1 serving = 118.3 g. Intermediate scores for vegetable intakes are calculated as follows: score = vegetable intake (g/d)*10/(5*118.3). An intake of 300 g will therefore translate to a score of 5.1 for the vegetable component

^2^ Fruit juice are not included in the fruit component; 1 serving = 118.3 g.

^3^ 100%-fruit juices are included in the sugar-sweetened beverages and fruit juice component; 1 serving is 226.8 g

^4^ 1 serving = 28.35 g

^5^ 1 serving of red meat is 113.4g and 1 serving of processed meat is 42.5g

^6^ Sex-specific cut-offs were computed for deciles

^7^ 1 drink of alcohol contains 10 g of ethanol

**Supplementary Figure S1. Participants flowchart, NutriNet-Santé cohort (2009-2020) – SAPRIS-SERO**

**
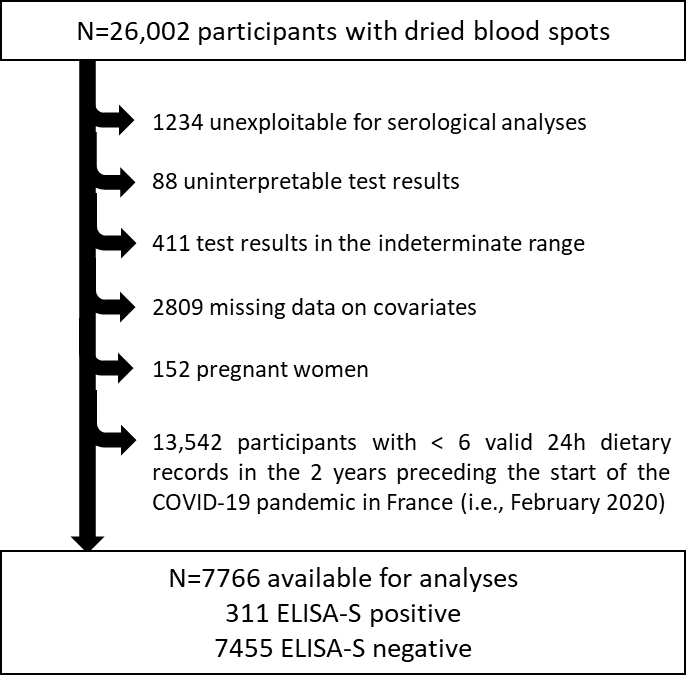
**

**Supplementary Table S1. Nutritional intakes, food group consumption and SARS-CoV-2 infection (ELISA-S), sensitivity analyses, NutriNet-Santé cohort (2009-2020) – SAPRIS-SERO.**

|  | **Per 1-SD increment** | | |
| --- | --- | --- | --- |
|  | **SD** | **OR (95%CI)^a^** | **P** |
| **Macronutrients** |  |  |  |
| Energy, without alcohol (kcal/d) | 403.6 | 1.00 (0.87,1.16) | 1.00 |
| Alcohol (g/d) | 10.7 | 1.00 (0.86,1.16) | 0.99 |
| Protein (g/d) | 18.3 | 1.00 (0.83,1.21) | 1.00 |
| Carbohydrates, total (g/d) | 52.4 | 1.05 (0.83,1.32) | 0.70 |
| Sugars (g/d) | 28.9 | 1.03 (0.88,1.22) | 0.68 |
| Fiber (g/d) | 7.0 | 0.81 (0.67,0.97) | **0.03** |
| Fiber, soluble (g/d) | 2.5 | 0.80 (0.67,0.96) | **0.01** |
| Fiber, insoluble (g/d) | 4.8 | 0.85 (0.71,1.01) | 0.06 |
| Fatty acids, total (g/d) | 22.2 | 0.96 (0.76,1.21) | 0.70 |
| Saturated fatty acids (g/d) | 11.0 | 1.09 (0.90,1.33) | 0.37 |
| Monounsaturated fatty acids (g/d) | 9.0 | 0.91 (0.76,1.10) | 0.32 |
| Polyunsaturated fatty acids (g/d) | 4.5 | 0.90 (0.77,1.06) | 0.21 |
| n-3 polyunsaturated fatty acids, total (g/d) | 0.88 | 0.90 (0.76,1.06) | 0.19 |
| n-3 linolenic acid (g/d) | 0.76 | 0.95 (0.81,1.11) | 0.52 |
| n-3 eicosapentaenoic acid (g/d) | 0.14 | 0.88 (0.76,1.02) | 0.09 |
| n-3 docosahexaenoic acid (g/d) | 0.16 | 0.87 (0.75,1.01) | 0.07 |
| n-6 polyunsaturated fatty acids, total (g/d) | 3.9 | 0.92 (0.79,1.07) | 0.27 |
| Cholesterol (mg/d) | 112.2 | 0.99 (0.85,1.15) | 0.87 |
| **Vitamins** |  |  |  |
| Vitamin A^b^ (µg/d) | 591.2 | 0.96 (0.84,1.10) | 0.53 |
| Vitamin B1 (mg/d) | 0.41 | 1.04 (0.92,1.18) | 0.53 |
| Vitamin B2 (mg/d) | 0.48 | 1.14 (0.99,1.31) | 0.06 |
| Vitamin B3 / PP (mg/d) | 5.6 | 1.03 (0.89,1.19) | 0.71 |
| Vitamin B5 (mg/d) | 1.3 | 1.06 (0.91,1.23) | 0.45 |
| Vitamin B6 (mg/d) | 0.48 | 0.91 (0.77,1.07) | 0.25 |
| Vitamin B9 (µg/d) | 92.6 | 0.83 (0.70,0.98) | 0.03 |
| Vitamin B12 (µg/d) | 3.6 | 0.97 (0.84,1.12) | 0.69 |
| Vitamin C (mg/d) | 48.6 | 0.86 (0.75,0.99) | **0.03** |
| Vitamin D (µg/d) | 1.8 | 0.94 (0.81,1.08) | 0.37 |
| Vitamin E (mg/d) | 4.2 | 0.89 (0.76,1.04) | 0.14 |
| Vitamin K (µg/d) | 109.0 | 0.86 (0.74,1.00) | 0.05 |
| **Minerals** |  |  |  |
| Selenium (µg/d) | 22.0 | 1.00 (0.87,1.15) | 0.98 |
| Zinc (mg/d) | 2.8 | 1.08 (0.91,1.28) | 0.38 |
| Calcium (mg/d) | 273.6 | 1.17 (1.01,1.36) | **0.03** |
| Magnesium (mg/d) | 137.8 | 1.00 (0.86,1.16) | 0.96 |
| Phosphorus (mg/d) | 382.2 | 1.09 (0.94,1.26) | 0.27 |
| Potassium (mg/d) | 752.6 | 0.92 (0.78,1.10) | 0.37 |
| Sodium (mg/d) | 894.3 | 0.94 (0.79,1.13) | 0.51 |
| Copper (mg/d) | 0.88 | 0.99 (0.86,1.13) | 0.84 |
| Iron (mg/d) | 4.6 | 0.97 (0.83,1.14) | 0.73 |
| Iodine (µg/d) | 111.9 | 1.03 (0.92,1.16) | 0.58 |
| Manganese (mg/d) | 2.2 | 0.97 (0.84,1.12) | 0.67 |
| **Food groups** |  |  |  |
| Fruit and vegetables (g/d) | 187.1 | 0.83 (0.71,0.97) | **0.02** |
| Legumes (g/d) | 23.5 | 0.93 (0.82,1.06) | 0.31 |
| Nuts, unsalted (g/d) | 10.7 | 1.01 (0.89,1.15) | 0.90 |
| Starchy foods^c^ (g/d) | 85.8 | 1.00 (0.88,1.15) | 0.95 |
| Red meat (g/d) | 26.0 | 0.90 (0.79,1.04) | 0.15 |
| Processed meat (g/d) | 22.3 | 1.08 (0.94,1.24) | 0.30 |
| Poultry (g/d) | 19.0 | 1.05 (0.94,1.18) | 0.38 |
| Eggs (g/d) | 15.7 | 0.94 (0.83,1.07) | 0.37 |
| Fish (g/d) | 23.8 | 0.92 (0.80,1.05) | 0.21 |
| Seafood (g/d) | 12.0 | 1.01 (0.89,1.16) | 0.83 |
| Dairy products (g/d) | 132.6 | 1.19 (1.06,1.32) | **0.003** |
| Cakes, cookies and pastries (g/d) | 37.5 | 0.99 (0.87,1.12) | 0.85 |
| Sweet products^d^ (g/d) | 50.2 | 1.06 (0.94,1.20) | 0.34 |
| Breakfast cereals (g/d) | 14.3 | 1.02 (0.91,1.14) | 0.73 |
| Unsweetened drinks (g/d) | 525.7 | 1.03 (0.92,1.16) | 0.63 |
| Sugary drinks (g/d) | 85.5 | 0.96 (0.85,1.09) | 0.50 |
| Alcoholic drinks (g/d) | 129.7 | 0.99 (0.85,1.14) | 0.85 |
| Added fat, plant origin (e.g., oil) (g/d) | 8.3 | 0.87 (0.75,1.00) | **0.048** |
| Added fat, animal origin (e.g., butter) (g/d) | 7.5 | 1.09 (0.96,1.24) | 0.17 |
| **Overall dietary quality** |  |  |  |
| Ultra-processed foods^e^ (%) | 6.7 | 0.95 (0.84,1.07) | 0.41 |

^a^ ELISA-S positive (n=311) compared to ELISA-S negative (n=7455) participants. Odds ratios and 95% confidence intervals per 1-SD increment obtained from multi-adjusted logistic regression models including sex (men/women), age, educational level (< high-school degree/ high-school degree/ undergraduate degree/ graduate degree), employment status (no professional activity prior to lockdown: unemployed, retired, homemaker/ short-time working / working outside home/ working from home/ student, trainee and other), smoking status (non-smoker, former smoker, smoker), presence of children and/or grandchildren aged under 18y at home (yes/no), residential area (rural area/ city < 20,000 inhabitants/ city ≥ 20,000 to 100,000 inhabitants/ city > 100,000 inhabitants), frequency of going out over the past week (never/ once/ 2 to 5 times/ 6 to 10 times/ > 10 times), prevalent chronic disease (cancer, cardiovascular disease, high blood pressure, diabetes, dyslipidemia; yes/no), geographical area (Paris Basin/ Centre-East/ East/ Mediterranean/ North/ West/ Paris region/ Southwest), BMI and physical activity level (high, moderate, low) prior to the March 2020 lockdown, month of blood draw (May-June/ July/ August-September-October), number of 24h dietary records, energy intakes (without alcohol, kcal/d), alcohol intakes (g/d; except for alcoholic drinks), a composite score reflecting the adherence to recommended protective behaviors when going out and the sPNNS-GS2 score reflecting the adherence to the French 2017 dietary recommendations (1).

^b^ Total vitamin A including retinol and beta-carotene, calculated as retinol equivalent (1mg retinol = 6mg beta-carotene)

^c^ Bread, pasta, rice, potatoes, starchy vegetables, etc.

^d^ Chocolate, sweets, honey, sugary desserts, etc.

^e^ Proportion of ultra-processed foods in the diet (based on the NOVA classification)

**Supplementary Figure S2.** Food group contributions to the intakes of nutrients, NutriNet-Santé cohort (2009-2020) – SAPRIS-SERO


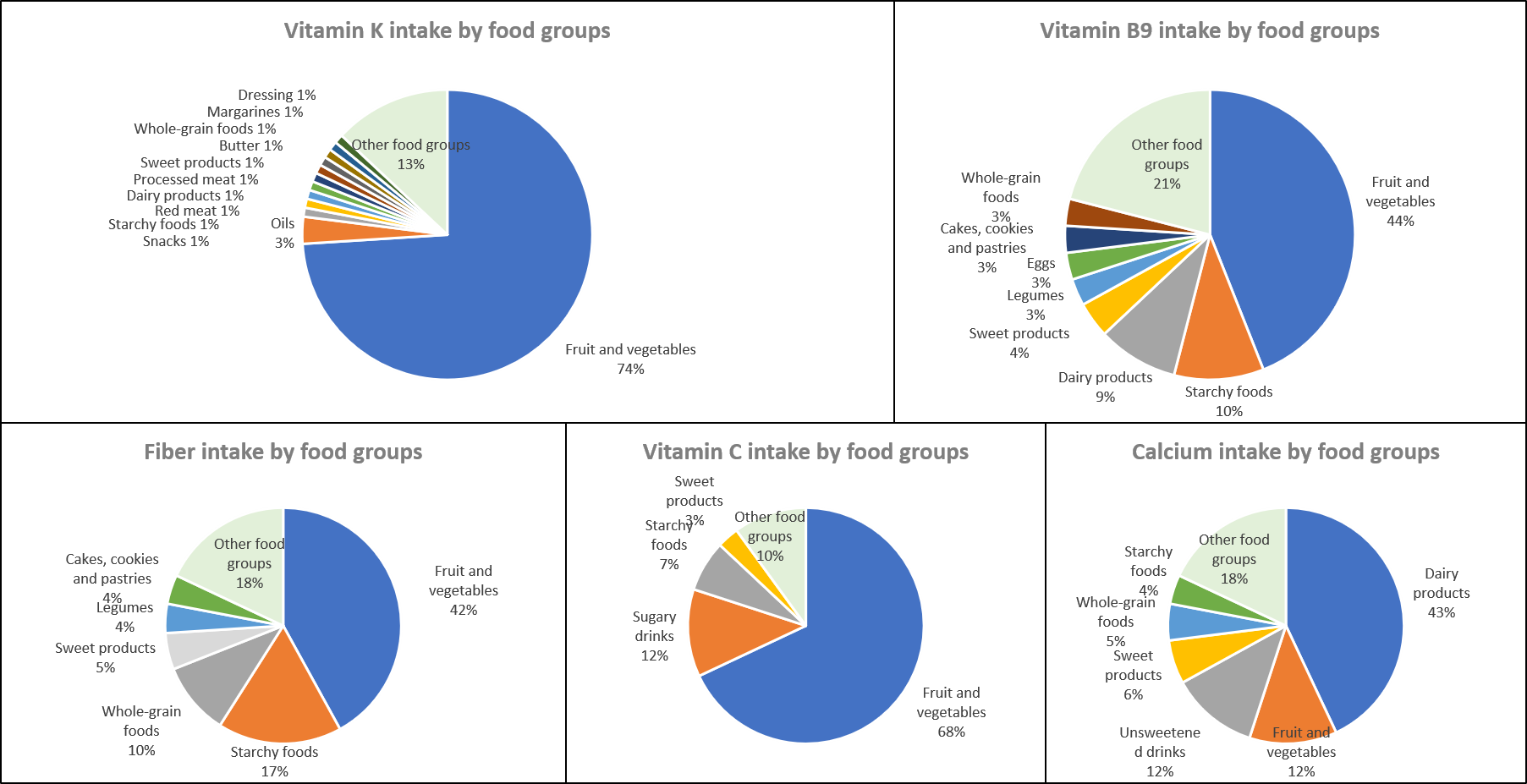


******* *for nutrients showing associations with the risk of SARS-Cov2 infection*

**Supplementary Figure S3. Nutritional intakes and SARS-CoV-2 infection (ELISA-S), nested case-control design, NutriNet-Santé cohort (2009-2020) – SAPRIS-SERO.** ELISA-S positive (n=311) compared to ELISA-S negative (n=1,244) controls, matched for age, sex and residential area. Odds ratios and 95% confidence intervals per 1-SD increment obtained from multi-adjusted logistic regression models including sex (men/women), age, educational level (< high-school degree/ high-school degree/ undergraduate degree/ graduate degree), employment status (no professional activity prior to lockdown: unemployed, retired, homemaker/ short-time working / working outside home/ working from home/ student, trainee and other), smoking status (non-smoker, former smoker, smoker), presence of children and/or grandchildren aged under 18y at home (yes/no), residential area (rural area/ city < 20,000 inhabitants/ city ≥ 20,000 to 100,000 inhabitants/ city > 100,000 inhabitants), frequency of going out over the past week (never/ once/ 2 to 5 times/ 6 to 10 times/ > 10 times), prevalent chronic disease (cancer, cardiovascular disease, high blood pressure, diabetes, dyslipidemia; yes/no), geographical area (Paris Basin/ Centre-East/ East/ Mediterranean/ North/ West/ Paris region/ Southwest), BMI and physical activity level (high, moderate, low) prior to the March 2020 lockdown, month of blood draw (May-June/ July/ August-September-October), number of 24h dietary records, energy intakes (without alcohol, kcal/d; except for energy), alcohol intakes (g/d; except for alcohol) and a composite score reflecting the adherence to recommended protective behaviors when going out. Total vitamin A including retinol and beta-carotene, calculated as retinol equivalent (1mg retinol = 6mg beta-carotene).

**
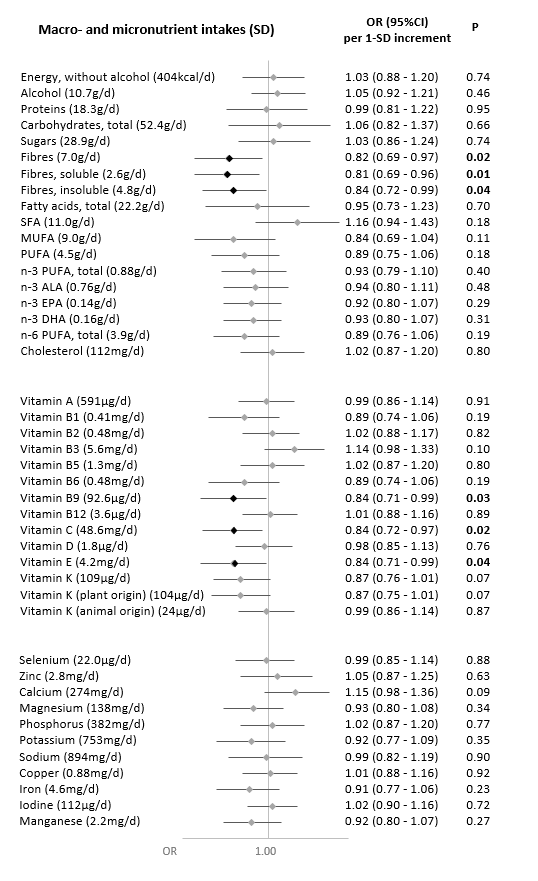
**

**Supplementary Figure S4. Food group consumption and SARS-CoV-2 infection (ELISA-S), nested case-control design, NutriNet-Santé cohort (2009-2020) – SAPRIS-SERO.** ELISA-S positive (n=311) compared to ELISA-S negative (n=1,244) controls, matched for age, sex and residential area. Odds ratios and 95% confidence intervals per 1-SD increment obtained from multi-adjusted logistic regression models including sex (men/women), age, educational level (< high-school degree/ high-school degree/ undergraduate degree/ graduate degree), employment status (no professional activity prior to lockdown: unemployed, retired, homemaker/ short-time working / working outside home/ working from home/ student, trainee and other), smoking status (non-smoker, former smoker, smoker), presence of children and/or grandchildren aged under 18y at home (yes/no), residential area (rural area/ city < 20,000 inhabitants/ city ≥ 20,000 to 100,000 inhabitants/ city > 100,000 inhabitants), frequency of going out over the past week (never/ once/ 2 to 5 times/ 6 to 10 times/ > 10 times), prevalent chronic disease (cancer, cardiovascular disease, high blood pressure, diabetes, dyslipidemia; yes/no), geographical area (Paris Basin/ Centre-East/ East/ Mediterranean/ North/ West/ Paris region/ Southwest), BMI and physical activity level (high, moderate, low) prior to the March 2020 lockdown, month of blood draw (May-June/ July/ August-September-October), number of 24h dietary records, energy intakes (without alcohol, kcal/d), alcohol intakes (g/d; except for alcoholic drinks) and a composite score reflecting the adherence to recommended protective behaviors when going out. Starchy foods: bread, pasta, rice, potatoes, starchy vegetables, etc.; sugary products: chocolate, sweets, honey, sugary desserts, etc.

**
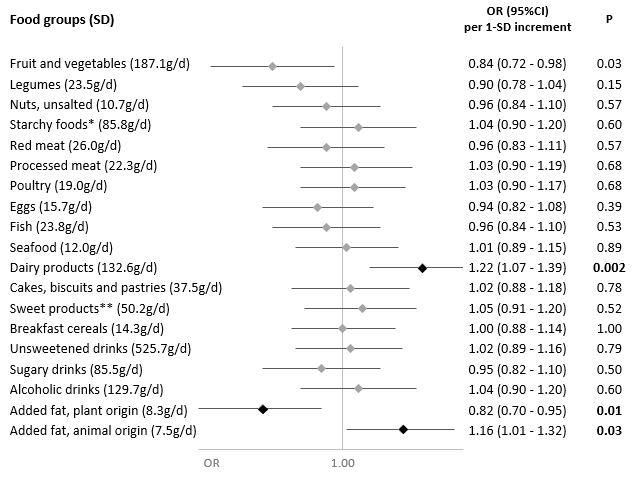
**
